# Effectiveness of cognitive interventions for adult surgical patients after general anaesthesia to improve cognitive functioning: A systematic review

**DOI:** 10.1111/jocn.16423

**Published:** 2022-06-22

**Authors:** Tracey Bowden, Catherine S. Hurt, Julie Sanders, Leanne M. Aitken

**Affiliations:** ^1^ School of Health Sciences City, University of London London UK; ^2^ St Bartholomew's Hospital Barts Health NHS Trust London UK; ^3^ The William Harvey Research Institute Queen Mary University London London UK

**Keywords:** cognitive dysfunction, cognitive intervention, general surgery, memory, postoperative cognitive complications, systematic review

## Abstract

**Aims and objectives:**

To examine the effectiveness of cognitive interventions after general anaesthesia to improve cognitive functioning.

**Background:**

The number of surgical procedures performed worldwide is large and growing. Postoperative cognitive dysfunction is a common complication associated with poor postoperative outcomes. A variety of cognitive interventions have been developed to maintain or improve cognitive function in one or more cognitive domains. Cognitive interventions have shown to be effective in healthy older populations, those with mild cognitive impairment, and those with heart failure. The impact of cognitive interventions in surgical patients after general anaesthesia is a relatively new focus of research and is therefore less well established.

**Methods:**

Seven bibliographic databases were searched in relation to ‘surgery’ and ‘cognitive interventions’; no date or language limits were imposed. Studies including adult patients who were scheduled for, or who had undergone surgery under general anaesthesia, had a baseline cognitive assessment using a validated measurement, and had engaged with any cognition‐based intervention were included. Full‐text review for inclusion, quality assessment and data extraction were undertaken independently by two authors. This study is reported in accordance with the Preferred Reporting Items for Systematic reviews and Meta‐Analyses guidelines.

**Results:**

A total of 550 papers were identified for possible inclusion, of which nine met the inclusion criteria and were included in the review. The majority were randomised controlled trials (RCTs) (*n* = 6 [66.7%]). Four studies used computerised cognitive interventions, while five used traditional cognitive interventions. Most of the studies used multi‐domain cognitive training focusing on two or more domains (*n* = 7 [77.8%]) while two studies used single‐domain cognitive training. Memory (*n* = 7 [77.8%]) and attention (*n* = 5 [55.6%]) were the cognitive domains most often targeted during the intervention.

**Conclusions:**

The use of cognitive interventions demonstrated some efficacy in improving cognitive function after general anaesthesia, particularly those targeting memory.

**Relevance for clinical practice:**

The findings of this review suggest that cognitive interventions show promise at improving cognitive performance in patients with POCD and could be usefully implemented in clinical practice to improve patient outcomes.

**What does this paper contribute to the wider global community?**

- Cognitive interventions are effective in healthy older populations, those with mild cognitive impairment and those with heart failure; however, the impact of cognitive interventions in surgical patients after general anaesthesia is less well established. This review suggests that cognitive interventions show promise at improving cognitive performance in patients with POCD and could be usefully implemented in clinical practice to improve patient outcomes.
- This review highlighted the paucity of research into intervention acceptability.

## 1 INTRODUCTION

Postoperative cognitive dysfunction (POCD), a decline in cognitive function measured objectively before and after surgery (Belrose & Noppens, 2019), is a common postoperative compilation. The number of surgical procedures performed worldwide is large, growing and ageing, with an estimated total of 313 million procedures performed annually (Meara et al., 2015). Older patients present with complex medical needs which can significantly impact postoperative outcomes, including cognitive function. The health and economic burdens associated with postoperative cognitive dysfunction are therefore likely to increase (Safavynia & Goldstein, 2019).

### 1.1 Background

Reports of postoperative cognitive impairment date back to the advent of cardiopulmonary bypass (Bedford, 1955; Hessel, 2014). While technological advancements and refinement of surgical techniques have resulted in a reduction in mortality, cognitive impairment remains a common postoperative complication (Berger et al., 2015). The incidence of POCD varies depending on factors such as the group of patients being studied, the definition of POCD used, the neuropsychological tests employed, the timing of testing and the type of surgery (Kotekar & Nagaraj, 2018; Rundshagen, 2014). The incidence of POCD decreases over time, with the highest rates occurring in the weeks following surgery; 30% to 80% of patients experience this complication after cardiac surgery, and 30% to 50% experience POCD after non‐cardiac surgery (Kotekar & Nagaraj, 2018; Sharipova, 2017). Manifestations can be subtle and vary depending on the cognitive domains affected, although impairments in memory, language skills and attention, as well as subjective complaints of difficulty thinking and concentrating, are commonly reported (Nelli et al., 2019). POCD is usually transient; however, it is associated with poor postoperative outcomes including prolonged length of hospital stay, increased morbidity and mortality, and reduced quality of life, resulting in a significant burden on the healthcare system (Cropsey et al., 2015; Steinmetz, 2009).

Methodological issues related to POCD, including the lack of consensus on how to define it, have been well documented (Funder et al., 2010). To address these challenges, recommendations for the nomenclature of cognitive change associated with anaesthesia and surgery were developed to align with clinical diagnostic criteria of neurocognitive disorders already in use (Evered et al., 2018). Postoperative neurocognitive disorders are subdivided according to the onset of impairment: up to 30‐days (delayed neurocognitive recovery) or 12‐months after surgery (postoperative neurocognitive disorder [POCD]). Use of the term ‘POCD’ remains in parentheses to help acknowledge the temporal relationship to surgery; its use is recommended for the transition period while the new nomenclature is integrated into practice. However, we use the term POCD to describe an objective postoperative decline in cognitive function to reflect the existing body of literature.

POCD has been extensively researched with many studies reporting that the cognitive domains of memory, attention, psychomotor speed and visuospatial ability are most frequently impaired (Ajtahed et al., 2019; Kok et al., 2017; Plaschke et al., 2013). Numerous pharmacological (Mathew et al., 2013; Ottens et al., 2014) and operative strategies (Hogan et al., 2013) have been explored in attempt to prevent POCD; however, limited clinically important improvements have been identified to date. Emerging evidence suggests that the use of cognitive interventions could lead to improved cognitive outcomes after surgery. For example, a recent study investigating the effect of cognitive prehabilitation, using preoperative computerised cognitive training, in 251 older adults scheduled for major non‐cardiac, non‐neurological surgery, resulted in a reduction in the incidence of delirium in the intervention group, as compared with controls (Humeidan et al., 2021).

Cognitive interventions (encompassing cognitive training, cognitive stimulation, and cognitive rehabilitation) are based on the principle that the inherent plasticity of the brain allows some degree of recovery following injury, meaning that the brain has the capacity to recover and make new connections following injury (Strobach & Karbach, 2021). Therefore, the aim of cognitive interventions is to enhance cognitive function. Cognitive training, the most frequently reported approach, involves repetitive training on specific tasks that can target a specific cognitive domain (Huntley et al., 2015). This can be achieved through traditional interventions (verbal, and pen‐and‐paper) or computerised interventions. Cognitive stimulation involves engaging individuals or groups in cognitively stimulating activities, for example board games, words searches or discussions related to current news stories, with the aim of improving general cognitive and social function (Deemer et al., 2020). Cognitive rehabilitation focuses on the development of strategies to improve functional ability following a specific impairment, with the aim of maintaining independence in activities of living (Deemer et al., 2020; Huntley et al., 2015).

Cognitive interventions have been shown to be effective in healthy older populations (Kelly et al., 2014), and those with mild cognitive impairment (Li et al., 2011; Martin et al., 2011) and heart failure (Ellis et al., 2014) with reported improvements in overall cognition and subjective cognition (Kelly et al., 2014; Li et al., 2011). It is therefore plausible that cognitive interventions may improve cognitive function following surgery and reduce the incidence of POCD. The impact of cognitive interventions in surgical patients after general anaesthesia is a relatively new focus of research and is therefore less well established. Thus, the aim of this systematic review was to determine whether, in surgical patients after general anaesthesia, the use of cognitive interventions compared to standard care, improves postoperative cognitive function.

## 2 METHODS

This study is reported in accordance with the Preferred Reporting Items for Systematic reviews and Meta‐Analyses guidelines (PRISMA) (Moher et al., 2009) (see supporting information file 1). This review was registered on PROPSERO (CRD42020184161).

### 2.1 Search terms and strategies

Seven bibliographic databases (MEDLINE, Cumulative Index to Nursing and Allied Health Literature [CINAHL], Embase, PsycINFO, Cochrane Library, ProQuest and Open Grey) were searched from inception to November 2021 for relevant literature by one author (TB). No language limits were imposed. In collaboration with an information specialist, searches were devised without methodological search filters that would limit results to specific study designs. Subject headings and keywords were used in the search in relation to two concepts: surgery and cognitive interventions, with the concepts combined using ‘AND’ for the final search (see Supplementary File 2 Table S1 for search syntax). A supplementary hand search of reference lists of the included papers and relevant systematic reviews was conducted.

Eligible studies included (a) adult patients (≥18‐years of age) who have had or are scheduled for surgery under a general anaesthetic and have had a baseline measure of cognitive function, (b) any cognition‐based intervention aimed at improving cognitive function after general anaesthesia, (c) an objective measure of cognitive function, measured before and after the cognitive intervention, and at least 7‐days postoperatively with either: an objective measure of global cognition using a validated instrument, for example the Montreal Cognitive Assessment (MoCA), or Mini‐Mental State Examination (MMSE), but not limited to these instruments, *or* an objective measure assessing a specific cognitive domain such as executive function, attention, processing speed, memory and language, using a validated instrument, and (d) primary research. Patients who have had or are scheduled for neurological surgery were excluded. Reasons for exclusion are presented in the Supplementary File 2 (Table S2). Titles and abstracts of identified articles were subject to blind independent review by two authors (TB and either CSH, JS, or LMA); conflicts were resolved through discussion in the pair. Full text of eligible papers was reviewed using a similar process.

### 2.2 Search outcomes

**FIGURE 1:**
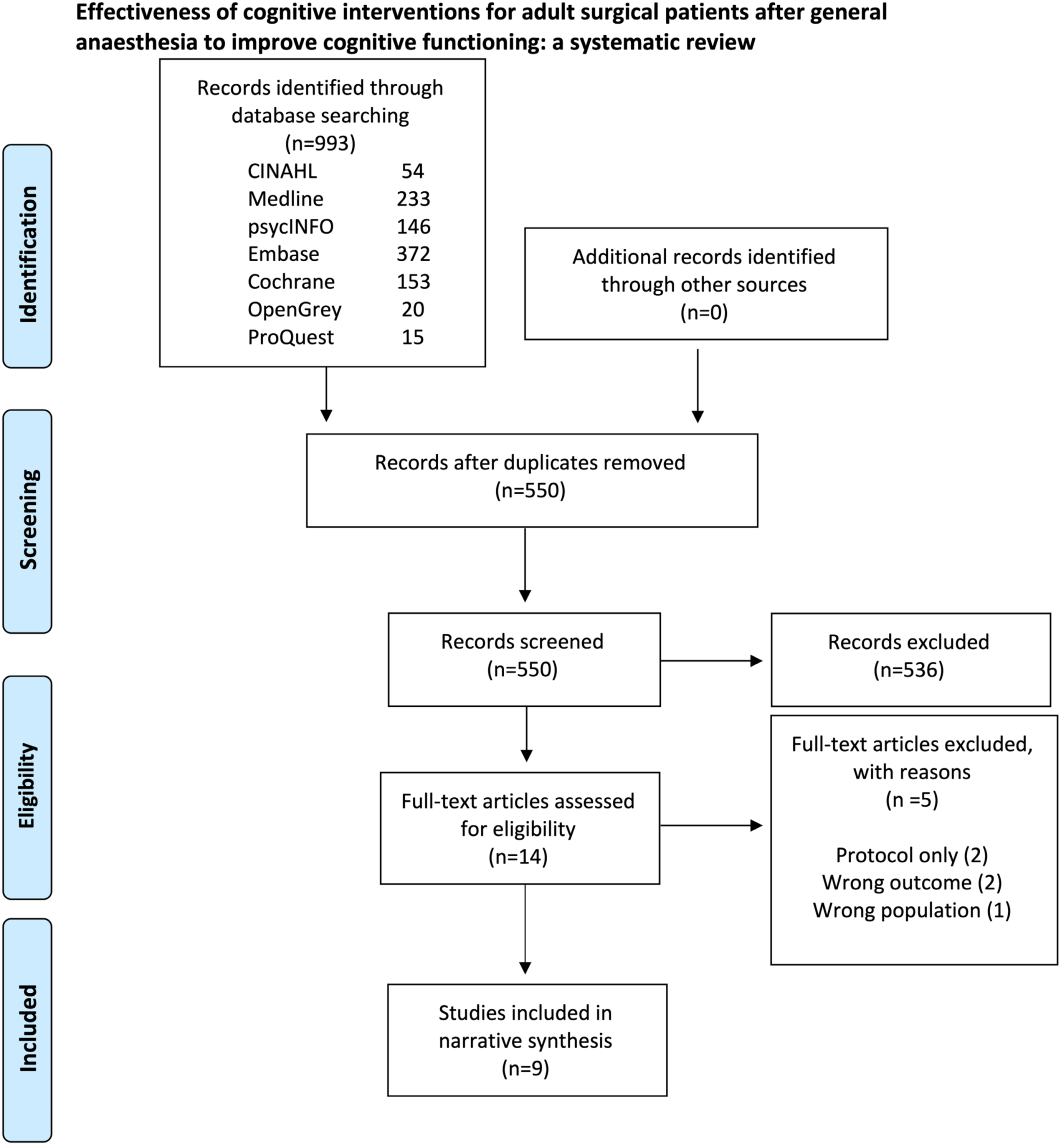
PRISMA flow diagram. [Colour figure can be viewed at wileyonlinelibrary.com]

A total of 550 papers were identified for possible inclusion (Figure 1). Though no language limits were imposed, the search only yielded papers published in English. Of these, 14 were retained for full‐text independent assessment. Overall, nine papers were included for data synthesis. A manual search of the reference lists did not yield any further papers. Inter‐rater reliability for inclusion was excellent (Kappa [*κ*] statistic 0.929–0.95).

### 2.3 Quality appraisal

Quality assessment was performed by two authors (TB and either CSH, JS, or LMA) using the Critical Appraisal Skills Programme [CASP] templates for cohort studies (2018a) and randomised controlled trials [RCTs] (2018b). Disagreements were resolved through discussion until consensus was achieved. Methodological quality was assessed in relation to selection bias, detection bias, attrition bias, other bias (all studies), reporting bias (RCTs) and confounding bias (non‐RCTs). The agreed quality assessment information was then used to generate a risk of bias graph and risk of bias summary (Review Manager (RevMan) [Computer program], 2014) for RCTs and non‐RCTs. The assessment score of each study was subsequently aggregated to develop a score for each paper as a percentage of the number of met criteria out of the total number of applicable criteria (Al‐Amer et al., 2022; Villarosa et al., 2019). Studies with a score of less than 60% were considered poor quality, 60%–79% were considered moderate quality, and 80% or greater were considered high quality. To ensure potentially valuable results in all studies were included, no studies were excluded on the basis of quality.

### 2.4 Data abstraction

A standardised proforma was developed and used to collect the following information about the study, population, intervention, comparison and outcome, from each paper included at the full‐text stage. Study details included information such as author, date of publication, title, study design, study aims, recruitment methods, length of follow‐up and allocation of treatment. Population information included the setting, country, type of surgery patients had undergone, mean age, gender, presence of any significant comorbidities and years of education. Intervention information included timing and duration (including preoperative or postoperative), type of intervention (computerised or traditional), the cognitive domains targeted and details of the individuals delivering the intervention (including professional background and training). Comparison groups were described, and results were recorded for cognitive outcomes. Outcome information included the measurement tool or instrument used, timing of outcome measures and the results for each group, and for each outcome at each time point. Data extraction was performed by two authors (TB and either CSH, JS, or LMA) with disagreements resolved through discussion until consensus was achieved.

### 2.5 Synthesis

The key features and findings of included studies were evaluated and summarised by Author 1, then discussed with the review team until agreement was reached. Due to heterogeneity of the included studies, meta‐analysis was not performed. Instead, results were summarised using descriptive statistics, tables and narrative synthesis.

## 3 RESULTS

### 3.1 Study characteristics and quality appraisal

Studies were conducted across three continents: Asia (*n* = 6), Europe (*n* = 1) and North America (*n* = 2), and the majority were RCTs (*n* = 6 [66.7%]). Studies included a total of *n* = 511 patients. There was significant variation in the study samples in relation to the type of surgery patients had undergone: cardiac (*n* = 4), gastrointestinal (*n* = 1), orthopaedic (*n* = 2) and pulmonary (*n* = 2). The intervention characteristics, study characteristics and main results are shown in Tables 1, 2, 3 and Supplementary File 2 Table S3.

**TABLE 1:** Intervention characteristics of included studies

Study *Primary author (year)*	Aim of intervention	Intervention *Type*, *timing*, *control group(s)*
Ajtahed et al. (2019)	To assess the efficacy of using CCRT as a supplementary treatment to RCR in patients after CABG surgery in terms of both cognitive functioning and QoL.	Computerised training with a commercially available program: Maghzineh® 24‐×‐20‐min sessions over 8‐weeks, postoperatively (it is unclear which week sessions commenced) Control (usual care) and active controls (sham version of CCT)
Carbone et al. (2019)	To assess the efficacy of working memory training in improving cognitive function and mood, or emotional functioning, in adults undergoing major surgery.	Working memory training 3 sessions (30‐40‐min each) completed within 2‐weeks postoperatively. Active control (alternative activities)
Cheng et al. (2012)	To determine the effects of a daily, individual‐based, cognitive stimulation intervention in older hospitalised patients undergoing elective TKR and/or THR surgery.	Cognitive stimulation (orientating communication and cognitively stimulating activities) Daily 20‐ to 30‐min sessions (most patients receiving 6‐days), postoperatively (while hospitalised) Control (usual care)
De Tournay‐Jetté et al. (2012)	To assess the efficacy of attentional training and memory training to enhance cognitive function in patients aged 65‐years and older who underwent CABG surgery.	Memory (MoL and story generation) and attention (dual task training: auditory and visual) training 8‐×‐50‐min sessions, delivered between the 6th and 10th week postoperatively Control (usual care)
Eryomina et al. (2015)	To evaluate the effectiveness of using computer‐based stimulation programs in the correction of cognitive impairments in patients with coronary heart disease after CABG.	Computer‐based stimulation programs Daily 2‐min sessions, delivered within 10 days after surgery Control (usual care)
Kulason et al. (2018)	To investigate the beneficial effects of SCRA intervention on cognitive functions and mental health in the elderly Japanese population after thoracic surgery.	Simple calculation (arithmetic) and reading aloud 3–5 × 30‐min sessions per week for 12 weeks, postoperatively. Training commenced after baseline assessment at 7.7 ± 3.06 days postoperatively Control (usual care)
O'Gara et al. (2020)	To determine the feasibility and potential efficacy of a perioperative cognitive training program to reduce the incidence of postoperative delirium and POCD in older cardiac surgery patients.	Computerised training with a commercially available program: Lumosity 2 × 15‐min sessions per day from enrolment (≥10 days before surgery) until 4 weeks postoperatively Control (usual care)
Saleh et al. (2015)	To evaluate whether preoperative cognitive training could lower the incidence of early cognitive dysfunction in elderly patients 1 week after gastrointestinal surgery.	Memory training (MoL) 3 × 1‐h sessions (scheduled 1 day apart), preoperatively (while hospitalised) Control (usual care)
Song et al. (2019)	To analyse the effect of CCT on elderly lung transplant recipients.	Computerised training with commercially available program: Posit Science BrainHQ 5 × 40‐min sessions (4 tasks, 10 min per task) per week, for 8 weeks, commencing 4 weeks postoperatively Control (usual care)

Abbreviations: CCRT, computerised cognitive rehabilitation therapy; CABG, coronary artery bypass graft; CCT, computerised cognitive training; MoL, method of loci; QoL, quality of life; RCR, routine cardiac rehabilitation; SCRA, simple calculation and reading aloud; THR, total hip replacement; TKR, total knee replacement.

**TABLE 2:** Study characteristics of included studies

Study Primary author (year), country	Study population		Specific domains targeted^a^	Timing of assessment(s)
	Type of surgery, Number (IG/CG)	Mean age IG/CG Years (SD)		
Ajtahed et al. (2019), Iran	CABG, 25/22/25^b^	59.96 (15.20)/ 57.95 (9.76)/ 56.48 (12.73)^b^	Attention, working memory, inhibition.	Pre‐intervention (after surgery), Post‐intervention, 6‐month follow‐up.
Carbone et al. (2019), Italy	Partial or total arthroplasty of the knee, 18/16	69.50 (3.20)/ 69.69 (4.01)	Working memory.	Pre‐intervention (before surgery), immediately post‐intervention.
Cheng et al. (2012), Taiwan	TKR ± THR, 25/25	73.0 (6.3)/ 72.6 (5.1)	Global cognitive function.	Pre‐intervention (before surgery), Post‐intervention (discharge), One month follow‐up.
De Tournay‐Jetté et al. (2012), Canada	CABG, 13/13/18^c^	69.92 (3.93)/ 70.85 (4.51)/ 70.89 (4.44)^c^	Attention, memory.	Pre‐intervention (1 month after surgery), 2 months post operatively (between intervention sessions, experimental groups only), Follow‐ups: 3 and 6 months.
Eryomina et al. (2015), Russia	CABG, 37/37	60.0 (6.42)/ 60.5 (6.42)	Attention, visual memorization, countdown, visuospatial stimulation.	Pre‐intervention (after surgery), follow‐up: 12 days.
Kulason et al. (2018), Japan	Lung surgery, 6/4	69 (6.96)/ 68.75 (4.27)	Global cognitive function Executive functions	Pre‐intervention (7.7 ± 3.06 days after surgery), 3‐month follow‐up.
O'Gara et al. (2020), USA	Cardiac surgery (CABG ± valve), 20/20	70 (6)/69 (7)	Memory, attention, problem solving, flexibility, processing speed.	Pre‐intervention (enrolment), preoperative (on the day of surgery), day of discharge, Follow‐ups: 1, 3 and 6 months postoperatively.
Saleh et al. (2015), China	Gastro‐intestinal surgery, 69/72	71 (6)/70 (6)	Memory.	Pre‐intervention (before surgery), Follow‐up: one week after surgery.
Song et al. (2019), China	Lung transplant, 23/23	65.0 (6.2)/ 66.8 (4.7)	Attention, information processing speed, working memory.	Pre‐intervention (4 weeks after surgery), immediately after intervention, 12‐week follow‐up.

*Notes*: ^a^ as reported by the authors of the studies; ^b^IG/AC/CG, ^c^Attention‐memory (A‐M) group/Memory‐attention (M‐A) group/control.

Abbreviations: AC, active control; CG, control group; IG, intervention group; SD, standard deviation.

**TABLE 3:** Results of included studies by cognitive domain measured

Domain measured^a^	Study Primary author (year)	Outcome measure	Significant effect for intervention group^b^
Attention	De Tournay‐Jetté et al. (2012)	TMT‐A	=
		TMT‐B	=
	Eryomina et al. (2015)	Schulte's tables	=
		Mattis dementia scale counting forward	=
	Saleh et al. (2015)	Digit span (forwards and backwards)	=
		TMT‐A	=
		TMT‐B	=
	Song et al. (2019)	TMT‐A	=
		TMT‐B	=
Divided attention	Ajtahed et al. (2019)	UFoVT	=
Selective attention	Ajtahed et al. (2019)	Flanker test	=
	De Tournay‐Jetté et al. (2012)	Stroop	+
Sustained attention	Ajtahed et al. (2019)	Continues performance test	=
	De Tournay‐Jetté et al. (2012)	WAIS‐R digit symbol	+
	Song et al. (2019)	Digit symbol test	=
Memory	De Tournay‐Jetté et al. (2012)	Logical memory subset of Rivermead battery	=
		RAVLT	+
	Eryomina et al. (2015)	10 word memory task	+
	Saleh et al. (2015)	BVMT‐R delayed recall	=
		BVMT‐R discrimination index	=
Verbal memory	Song et al. (2019)	Verbal fluency	+
Visual recognition memory	Eryomina et al. (2015)	Spontaneous visual memorisation (word list)	+
		Spontaneous visual memorisation (delayed recall)	+
	Saleh et al. (2015)	BVMT‐R	+
Working memory	Ajtahed et al. (2019)	Digit span (forwards and backwards)	+
	Carbone et al. (2019)	Digit span (forwards)	+
		Digit span (backwards)	=
		RAVLT immediate recall	=
		RAVLT delayed recall	=
		CWMST	+
		CWMST intrusion errors	+
Psychomotor speed	Saleh et al. (2015)	SDMT	+
Processing speed	Song et al. (2019)	Word recognition test	=
Visuospatial analysis	Saleh et al. (2015)	Benton judgement of line orientation	=
Executive function	De Tournay‐Jetté et al. (2012)	Verbal fluency test	=
	Eryomina et al. (2015)	Clock drawing test	=
		Counting down	=
		Verbal fluency test	+
		FAB	=
	Kulason et al. (2018)	FAB	=
	Saleh et al. (2015)	Verbal fluency test	=
	Song et al. (2019)	Digit span (forward)	+
		Digit span (backwards)	=
Motor programming	Kulason et al. (2018)	FAB motor programming subscore	+
Global	Cheng et al. (2012)	MMSE	+
	Eryomina et al. (2015)	MMSE	+
	Kulason et al. (2018)	CBB^c^	=
		MMSE‐J	=
	O'Gara et al. (2020)	t‐MoCA	=

*Notes*: ^a^ as reported by the authors of the studies; ^b^ at final follow‐up; ^c^ measurement of processing speed, visual attention, visual learning and memory, and attention and working memory. Effect of intervention: + positive effect for intervention group, = no difference between groups, − negative effect for intervention group.

Abbreviations: BVMT‐R, Brief Visuospatial Memory Test–Revised; CBB, Cogstate Brief Battery; CWMST, Categorization Working Memory Span Task; FAB, frontal assessment battery; MMSE, Mini‐Mental State Examination; MMSE‐J, Mini‐Mental State Examination–Japanese; RAVLT, Rey Auditory and Verbal Learning Test; SDMT, Symbol Digit Modalities Test; t‐MoCA, Telephonic Montreal Cognitive Assessment; TMT‐A, trail making test part A; TMT‐B, trail making test part B; UFoVT, useful field of view test; WAIS‐R, Wechsler Adult intelligence Scale–Revised.

Eight studies recruited participants from a single centre (Ajtahed et al., 2019;Carbone et al., 2019; Cheng et al., 2012; Eryomina et al., 2015; Kulason et al., 2018; O'Gara et al., 2020; Saleh et al., 2015; Song et al., 2019), while one recruited from multiple sites (De Tournay‐Jetté et al., 2012). Of the eight single centre studies, six were RCTs (Carbone et al., 2019; Cheng et al., 2012; Kulason et al., 2018; O'Gara et al., 2020; Saleh et al., 2015; Song et al., 2019) and two were non‐RCTs (Ajtahed et al., 2019; Eryomina et al., 2015). Further, 3 of the RCTs were pilot studies (Carbone et al., 2019; Cheng et al., 2012; Kulason et al., 2018) and two were feasibility studies (O'Gara et al., 2020; Song et al., 2019); therefore, sample sizes were generally small, ranging from 12 to 141 participants. In all studies, experimental and control groups were run concurrently with either two (Carbone et al., 2019; Cheng et al., 2012; Eryomina et al., 2015; Kulason et al., 2018; O'Gara et al., 2020; Saleh et al., 2015; Song et al., 2019) or three groups (Ajtahed et al., 2019; De Tournay‐Jetté et al., 2012). The majority of the studies compared the intervention with usual care (*n* = 7 [77.8%]) (Cheng et al., 2012; De Tournay‐Jetté et al., 2012; Eryomina et al., 2015; Kulason et al., 2018; O'Gara et al., 2020; Saleh et al., 2015; Song et al., 2019), while one used an active control group (Carbone et al., 2019) and another used both an active control group and a usual care group (Ajtahed et al., 2019).

Cardiac studies included slightly younger participants, with mean age extending from 60 to 70 years of age, while mean age in the other cohorts ranged from 65 to 73 years of age (Ajtahed et al., 2019; De Tournay‐Jetté et al., 2012; Eryomina et al., 2015; O'Gara et al., 2020). Orthopaedic studies included considerably lower proportions of males (16% to 38.9%) in comparison with the other surgical cohorts (50% to 76.9%) (Carbone et al., 2019; Cheng et al., 2012). The majority of studies included patients with any educational level (*n* = 7 [77.8%]) (Ajtahed et al., 2019; Carbone et al., 2019; Cheng et al., 2012; De Tournay‐Jetté et al., 2012; Eryomina et al., 2015; Hanling et al., 2016; Kulason et al., 2018; Saleh et al., 2015); however, one study included only those who had obtained an educational level of at least high school or equivalent (O'Gara et al., 2020) (Supplementary File 2 Table S4). Finally, five studies excluded patients based on their baseline cognition (Supplementary File 2 Table S5): four studies excluded patients with a Mini‐Mental State Examination (MMSE) of less than or equal to 23 (Saleh et al., 2015), 24 (De Tournay‐Jetté et al., 2012; Song et al., 2019) or 25 (Carbone et al., 2019). Two studies used the Montreal Cognitive Assessment (MoCA); however, the exclusion scores varied from MoCA <10 (O'Gara et al., 2020) to MoCA ≤26 (Song et al., 2019).

Measurement of cognitive function was achieved through a variety of domain‐specific measures in seven of the studies (Ajtahed et al., 2019; Carbone et al., 2019; De Tournay‐Jetté et al., 2012; Eryomina et al., 2015; Kulason et al., 2018; Saleh et al., 2015; Song et al., 2019), with the remaining two opting for measures of global cognition (Cheng et al., 2012; O'Gara et al., 2020). The digit span tests (forwards and backwards) were the most commonly used (*n* = 4 [44.4%]), followed by the MMSE (*n* = 3 [33.3%]) and the Trail Making Tests (TMT, *n* = 3 [33.3%]). Follow‐up periods varied, ranging from immediately post cognitive intervention (Carbone et al., 2019; Kulason et al., 2018) to 6 months post cognitive intervention (Ajtahed et al., 2019; De Tournay‐Jetté et al., 2012; O'Gara et al., 2020).

The cognitive interventions that patients received were varied in relation to the timing of surgery. Two started cognitive training preoperatively (O'Gara et al., 2020; Saleh et al., 2015), whereas seven studies started training postoperatively (Ajtahed et al., 2019; Carbone et al., 2019; Cheng et al., 2012; De Tournay‐Jetté et al., 2012; Eryomina et al., 2015; Kulason et al., 2018; Song et al., 2019). The commencement of postoperative training ranged from the immediate postoperative period (Cheng et al., 2012) to 4 weeks after surgery (Song et al., 2019). Finally, the duration of the interventions ranged from only three sessions scheduled 1 day apart (Saleh et al., 2015), to 12 weeks (Kulason et al., 2018).

Four of the studies used computerised cognitive interventions (Ajtahed et al., 2019; Eryomina et al., 2015; O'Gara et al., 2020; Song et al., 2019) while five used traditional cognitive interventions (Carbone et al., 2019; Cheng et al., 2012; De Tournay‐Jetté et al., 2012; Kulason et al., 2018; Saleh et al., 2015). Most of the studies used multi‐domain cognitive training focusing on two or more domains (*n* = 7 [77.8%]) while two studies used single‐domain cognitive training (Carbone et al., 2019; Saleh et al., 2015). Memory (*n* = 7 [77.8%]) and attention (*n* = 5 [55.6%]) were the cognitive domains most often targeted during the intervention.

**FIGURE 2:**
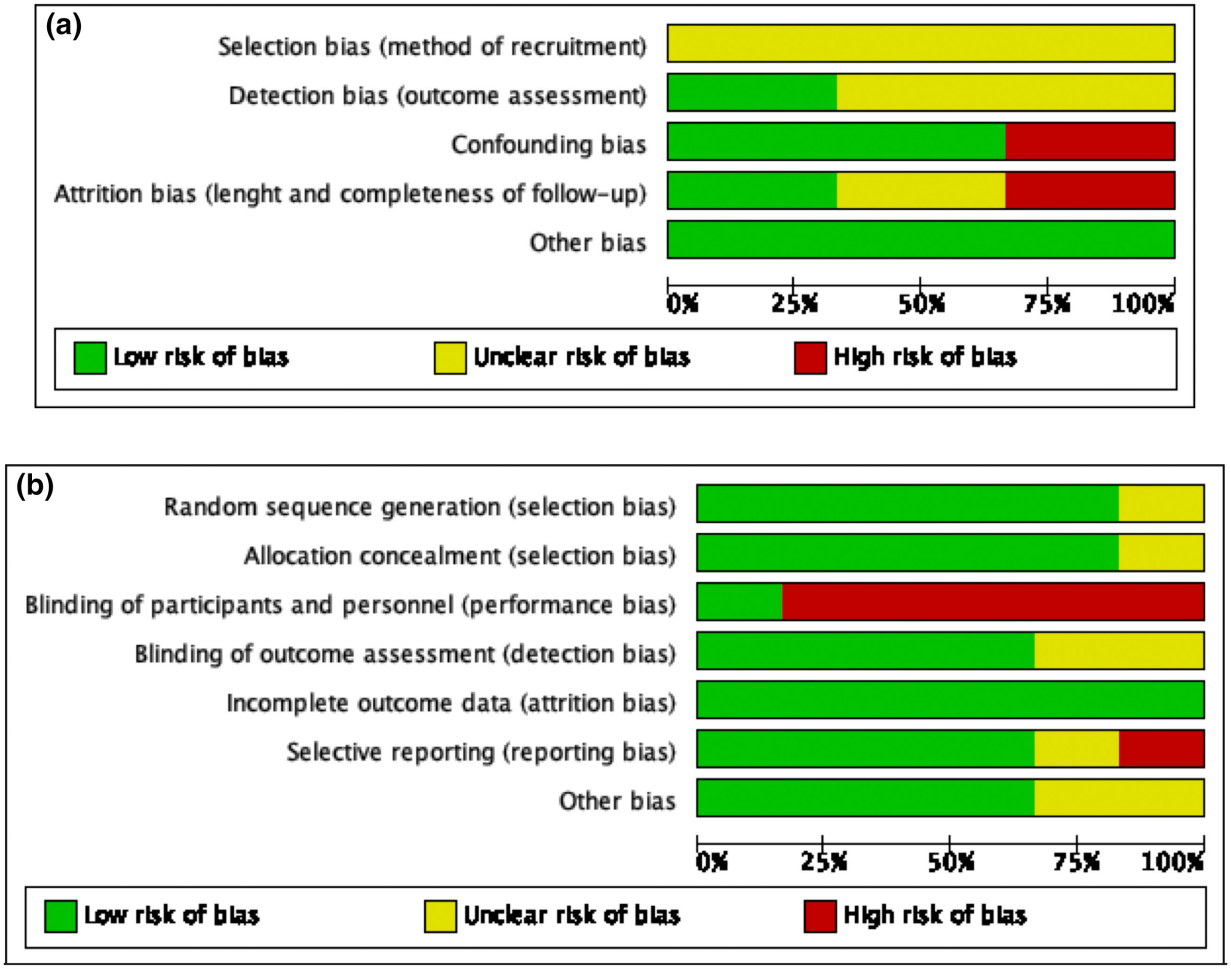
Risk of bias across studies. Review authors' judgements about each risk of bias item presented as percentages across all included studies: (a) non‐RCTs, (b) RCTs [Colour figure can be viewed at wileyonlinelibrary.com]

The risk of bias was greater in the non‐RCTs (Figure 2a, Supplementary File 2 Figure S1a). Of the three included non‐RCTs, one was assessed to be high quality (De Tournay‐Jetté et al., 2012) and two were of poor quality (Ajtahed et al., 2019; Eryomina et al., 2015). Methodological limitations included all three studies lacking details of patient recruitment (selection bias) (Ajtahed et al., 2019; De Tournay‐Jetté et al., 2012; Eryomina et al., 2015) and two studies lacking details of outcome assessors (detection bias) (Ajtahed et al., 2019; Eryomina et al., 2015); one of the studies also lacked details about the loss of participants during the course of the study (attrition bias) (Eryomina et al., 2015). Further, one study reported a high attrition rate (51%) at 6 months follow‐up (Ajtahed et al., 2019) and another lacked details of potential confounders (confounding bias) (Eryomina et al., 2015). Importantly, one study failed to provide any details of the cognitive intervention used (Eryomina et al., 2015). Two of the included RCTs were of high quality (O'Gara et al., 2020; Saleh et al., 2015), three were of moderate quality(Cheng et al., 2012; Kulason et al., 2018; Song et al., 2019), and one was of poor quality (Carbone et al., 2019). The greatest risk of bias across the RCTs was related to detection bias; specifically in five of the six RCTs (Carbone et al., 2019; Cheng et al., 2012; Kulason et al., 2018; O'Gara et al., 2020; Song et al., 2019), participants and investigators were not blinded to the intervention participants were allocated (Figure 2b, Supplementary File 2 Figure S1b). It is unclear whether outcome assessors were blinded to the intervention in two of the RCTs (Carbone et al., 2019; Kulason et al., 2018).

### 3.2 Computerised cognitive interventions

The effectiveness of computerised cognitive interventions was examined in four of the studies (Table 1) (Ajtahed et al., 2019; Eryomina et al., 2015; O'Gara et al., 2020; Song et al., 2019). Three studies employed commercially available programmes: Maghzineh (Ajtahed et al., 2019), Lumosity (O'Gara et al., 2020) and Posit Science BrainHQ (Song et al., 2019). The remaining study investigating computerised cognitive interventions did not provide details of the intervention (Eryomina et al., 2015). All the computerised cognitive interventions targeted the domains of memory and attention with specific tasks aimed at improving function within these domains. Significant improvement in memory was observed in trained participants when compared to control groups in three of the studies, specifically verbal memory (Song et al., 2019), visual recognition memory (Eryomina et al., 2015) and working memory (Ajtahed et al., 2019); however, there were no differences observed in any of the measures of attention.

### 3.3 Traditional cognitive interventions

Of the nine included studies, five employed a variety of traditional cognitive interventions (Table 2). The Method of Loci (MoL) was used as a strategy for memory enhancement in two of the studies (De Tournay‐Jetté et al., 2012; Saleh et al., 2015); otherwise, there was no overlap in the cognitive interventions examined. Three studies targeted interventions towards memory improvement; all three reported a significant improvement within this domain (Carbone et al., 2019; De Tournay‐Jetté et al., 2012; Saleh et al., 2015). Two studies aimed to improve global cognitive functioning (Cheng et al., 2012; Kulason et al., 2018). Cognitive stimulation with orientating communication and cognitively stimulating activities resulted in an improvement in MMSE scores (Cheng et al., 2012). In contrast, simple calculation and reading aloud (SCRA) did not result in an increase in Mini‐Mental State Examination–Japanese version (MMSE‐J), Frontal Assessment Battery (FAB) or Cogstate Brief Battery (CBB) scores; however, an improvement was reported in the motor programming subscore of the FAB (Kulason et al., 2018). In addition to memory training, De Tournay‐Jetté et al. (2012) included training to improve attention with mixed results. Using the MoL for memory enhancement before surgery, one study reported an improvement in psychomotor speed 1 week postoperatively (Saleh et al., 2015).

### 3.4 Quality of life and related outcomes

Quality of life was measured in two studies (Ajtahed et al., 2019; Kulason et al., 2018); both reported significant improvements in the intervention group quality of life scores (Supplementary File 2 Table S3). Depression was measured with the Geriatric Depression Scale (GDS) at the same time as cognition in three studies (Carbone et al., 2019; Cheng et al., 2012; Kulason et al., 2018). Of the studies measuring depressive symptoms, two reported significant improvements in the intervention group depression scores (Supplementary File 2 Table S3) (Carbone et al., 2019; Kulason et al., 2018). GDS results were not reported in the remaining study measuring depression (Cheng et al., 2012).

## 4 DISCUSSION

In this systematic review examining the effectiveness of cognitive interventions after general anaesthesia, cognitive interventions were effective in improving various aspects of objective cognitive functioning, particularly those targeting memory. Factors considered when analysing the effectiveness of cognitive interventions included the target population, cognitive domains targeted, training structure and outcome measures assessed. Differences in mean age and gender are most likely explained by the type of surgery. One study included patients with at least high school education or equivalent (O'Gara et al., 2020), the rationale being to screen out pre‐existing cognitive deficits and to homogenise baseline cognitive abilities of the study population. However, numerous studies have reported that lower educational levels are predictive of postoperative cognitive dysfunction (Berger et al., 2015; Glumac et al., 2019), implying that those with lower educational levels should be prioritised for targeted intervention to improve cognitive function.

Most studies showed that a cognitive intervention produced improvement in one or more of the domains trained (Ajtahed et al., 2019; Carbone et al., 2019; Cheng et al., 2012; De Tournay‐Jetté et al., 2012; Eryomina et al., 2015; Saleh et al., 2015; Song et al., 2019), particularly memory. Of those targeting memory, most reported a significant improvement in one or more of the tests measuring this domain (Ajtahed et al., 2019; Carbone et al., 2019; De Tournay‐Jetté et al., 2012; Eryomina et al., 2015; Saleh et al., 2015; Song et al., 2019). While three of these studies reported improvement across all the neuropsychological tests measuring memory (Ajtahed et al., 2019; Eryomina et al., 2015; Song et al., 2019), three reported mixed results (Carbone et al., 2019; De Tournay‐Jetté et al., 2012; Saleh et al., 2015). Differing sensitivities of the measures is one possible explanation for these inconsistencies. The efficacy of working memory training following orthopaedic surgery resulted in significant improvement in trained participants when compared to controls in some of the tests measuring memory; however, no difference was noted in the Rey Auditory and Verbal Learning Test (RAVLT) (Carbone et al., 2019). While RAVLT is a sensitive test of verbal learning and memory, performance can be affected by age, education and synchrony (Lehmann et al., 2013; Schoenberg et al., 2006). These results also suggest limited transfer of effects within the domain.

As indicated previously, the domain of attention is frequently impaired following surgery. In contrast to memory, the interventions targeting this domain have not demonstrated improvement despite a similar dose of training (frequency, intensity and duration), with only one of the five studies targeting attention reporting any improvement. A possible explanation of this discrepancy could be that impairments within the domain of attention are secondary to problems in executive functioning, for example cognitive flexibility, planning and inhibition. Therefore, targeting attention will not necessarily address the underlying cognitive impairments. Interestingly, all four studies using computerised interventions reported no difference between the trained groups and controls (Ajtahed et al., 2019; Eryomina et al., 2015; O'Gara et al., 2020; Song et al., 2019). In contrast to our review findings, video game‐based training with BrainHQ (Leung et al., 2015; O'Brien et al., 2013) and Lumosity (Ballesteros et al., 2014) have resulted in significant improvements in attention in older adults who were deemed at risk of cognitive decline (Leung et al., 2015) and in healthy older adults (Ballesteros et al., 2014; O'Brien et al., 2013). Though comparisons between studies are challenging due to heterogeneity in study populations, outcome measures and training programs used, the improvements in attention are encouraging and warrant further investigation.

Two studies explored the feasibility of computerised interventions using commercially available programs: Lumosity (O'Gara et al., 2020) and BrainHQ (Song et al., 2019). Both exceeded their target of randomising at least 50% of eligible participants suggesting efficient screening procedures and sufficient interest within respective populations. However, adherence rates ranged from 6% in the immediate postoperative period (O'Gara et al., 2020) to 90% (Song et al., 2019). Differences might relate to training timing that varied from commencing at least 10 days preoperatively (O'Gara et al., 2020) to four weeks after surgery (Song et al., 2019). Interestingly, the duration of the training sessions was longer in the study with better adherence rates suggesting that other factors, for example the study population, timing of training, or the cognitive training program, affected adherence rates. These findings are consistent with results of two further studies not meeting our inclusion criteria where patient compliance was low in the preoperative period (Humeidan et al., 2021; Vlisides et al., 2019).

Only one study considered the acceptability of the cognitive intervention where participants completed a postoperative survey following completion of cognitive training (O'Gara et al., 2020). Despite low adherence rates, participants reported that the program was enjoyable and easy to use, as well as improvements in memory and thinking ability. These findings suggest participants are willing to engage with cognitive training; however, further investigation is warranted to determine optimum timing and dosage of training to improve adherence.

While both traditional and computerised interventions have resulted in improved cognitive outcome, computerised interventions offer several advantages over traditional cognitive interventions including its ease of implementation. They are cost‐effective and can be accessed from anywhere, and at any time, including the users' home. Importantly, computerised cognitive training enables real‐time performance assessment and feedback, allowing for the adjustment of application difficulty, thereby maximising the potential benefits to the user (Alnajjar et al., 2019). Further, the COVID‐19 pandemic has resulted in an increased reliance on technology and a surge in patients' uptake of remote health services (Hutchings, 2020), which may translate into increased interest and ability in undertaking computerised cognitive training, particularly in the older adult population.

Importantly, improved cognition was associated with other improvements including quality of life (Ajtahed et al., 2019; Kulason et al., 2018), anxiety (Carbone et al., 2019) and depression (Carbone et al., 2019; Kulason et al., 2018). Quality of life has been demonstrated to be highly correlated with morbidity, mortality and healthcare costs; therefore, interventions improving quality of life have the potential to reduce burden on caregivers and healthcare systems (Cantelmo & Stefanacci, 2016).

### 4.1 Limitations

This systematic review has a several limitations. First, no study was excluded based on quality assessment. This decision was made to ensure potentially valuable results were included in the final synthesis and to provide a wide body of evidence; however, this also had the effect of impacting the risk of bias. Methodological quality of included studies varied according to study design with a greater risk of bias in the non‐RCTs. Second, several feasibility and pilot studies were included; therefore, sample sizes were small and power was not assessed, potentially limiting the generalisability of findings. Finally, methodological heterogeneity meant meta‐analysis was not possible.

## 5 CONCLUSION

The use of cognitive interventions demonstrated some efficacy in improving cognitive function after general anaesthesia, particularly those targeting memory. Adherence rates varied depending on the timing of interventions, with particularly low rates in the immediate postoperative period. This review highlighted the paucity of research into intervention acceptability within the postoperative population. Our findings suggest that patients are willing to engage with cognitive training; however, further investigation is warranted to determine optimum timing and dosage of training to improve adherence, efficacy and acceptability.

## 6 RELEVANCE TO CLINICAL PRACTICE

The findings of the systematic review suggest that cognitive interventions show promise at improving cognitive performance in patients with POCD and could be usefully implemented in clinical practice to improve patient outcomes.

## AUTHOR CONTRIBUTION

Conceptualization, funding acquisition, investigation, project administration, visualisation, and writing–original draft: **Tracey Bowden**; conceptualization, funding acquisition, investigation, supervision, and writing–review and editing: **Catherine S Hurt, Julie Sanders and Leanne M Aitken**.

## CONFLICT OF INTEREST

None.

## PROSPERO REGISTRATION

This review was registered on PROPSERO (CRD42020184161) https://www.crd.york.ac.uk/prospero/display_record.php?ID=CRD42020184161

## Supporting information


Appendix S1

## Data Availability

Data sharing not applicable—no new data generated.
