# Supplementary material for: Effectiveness of cognitive interventions for adult surgical patients after general anaesthesia to improve cognitive functioning: A systematic review
Source: J Clin Nurs. 2022 Jun 22;32(13-14):3117–29. doi: 10.1111/jocn.16423 (PMC10946716; doi:10.1111/jocn.16423)
Supplement: Supplementary file 1 — Appendix S1 [file JOCN-32-3117-s001.docx]

**Effectiveness of cognitive interventions for adult surgical patients after general anaesthesia to improve cognitive functioning: A systematic review**

**(Supporting Information)**

**Figure S1 Risk of bias summary**. Review authors' judgements about each risk of bias item for each included study: (A) non-RCTs, (B) RCTs

**Table S1** Search syntax

**Table S2** Excluded studies and reasons for exclusion

**Table S3** Study characteristics of included studies

| **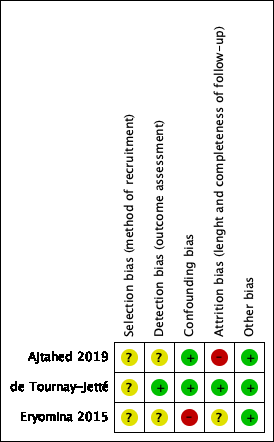** | **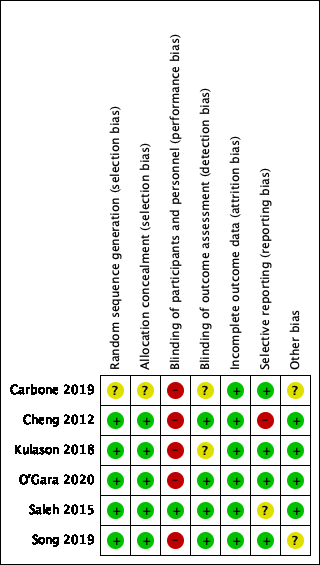** |
| --- | --- |
| **(A)** | **(B)** |

**Figure S1**

**Risk of bias summary**. Review authors' judgements about each risk of bias item for each included study: (A) non-RCTs, (B) RCTs

**Table S1**

**Search Syntax**

| **Database** | **Syntax** |
| --- | --- |
| CINAHL | ((MH “surgery, operative”) OR “surgery” OR “operation” OR “surgical procedure” OR “surgical treatment” OR “operative”) AND )(MH “cognitive remediation”) OR (MH “rehabilitation, cognitive”) OR “cognitive stimulation” OR “cognitive rehabilitation” OR “cognitive training” OR “cognitive retraining” OR “cognitive re-training” OR. “cognitive support” OR “memory function” OR “memory rehabilitation” OR “memory therapy” OR “memory aid*” OR “memory group” OR “memory stimulation” OR “memory strategy” OR “memory management”)  Searched via EBSCOhost on 16th September 2020 and updated on 17^th^ November 2021. No date or language limits were imposed, no exploding of MeSH.  54 results |
| Medline | (general surgery [MH: no exp] OR postoperative cognitive complications [MH: no exp] OR "surgical procedure” or “surgical treatment” OR “operative”) AND (cognitive remediation [MH: no exp] OR cognitive reserve [MH: no exp] OR “cognitive stimulation” OR “cognitive rehabilitation” OR “cognitive training” OR “cognitive retraining” OR “cognitive re-training” OR. “cognitive support” OR “memory function” OR “memory rehabilitation” OR “memory therapy” OR “memory aid*” OR “memory group” OR “memory stimulation” OR “memory strategy” OR “memory management”)  Searched via EBSCOhost on 16^th^ September 2020 and updated on 17^th^ November 2021. No date or language limits were imposed, no exploding of MeSH  233 results |
| psycINFO | (surgery/ OR “operation” OR “surgical procedure” OR “surgical treatment” OR “operative”) AND (cognitive remediation/ OR cognitive reserve/ OR cognitive rehabilitation/ OR “cognitive stimulation” OR “cognitive rehabilitation” OR “cognitive training” OR “cognitive retraining” OR “cognitive re-training” OR. “cognitive support” OR “memory function” OR “memory rehabilitation” OR “memory therapy” OR “memory aid*” OR “memory group” OR “memory stimulation” OR “memory strategy” OR “memory management”)  Searched via EBSCOhost on 16^th^ September 2020 and updated on 17^th^ November 2021. No date or language limits were imposed.  146 results |
| Embase | (general surgery/ OR “surgery” OR “operation” OR “surgical procedure” OR “surgical treatment” OR “operative”) AND (cognitive rehabilitation/ OR cognitive reserve/ OR cognitive remediation therapy/ OR “cognitive stimulation” OR “cognitive rehabilitation” OR “cognitive training” OR “cognitive retraining” OR “cognitive re-training” OR. “cognitive support” OR “memory function” OR “memory rehabilitation” OR “memory therapy” OR “memory aid*” OR “memory group” OR “memory stimulation” OR “memory strategy” OR “memory management”)  Searched via OVID on 16^th^ September 2020 and updated on 17^th^ November 2021. No date or language limits were imposed, no exploding of MeSH  372 results |
| Cochrane | (general surgery [MH: no exp] OR postoperative cognitive complications [MH: no exp] OR “surgery” OR “operation” OR “surgical procedure” OR surgical treatment” OR “operative”) AND (cognitive remediation [MH: no exp] OR cognitive reserve [MH: no exp] OR “cognitive stimulation” OR “cognitive rehabilitation” OR “cognitive training” OR “cognitive retraining” OR “cognitive re-training” OR. “cognitive support” OR “memory function” OR “memory rehabilitation” OR “memory therapy” OR “memory aid*” OR “memory group” OR “memory stimulation” OR “memory strategy” OR “memory management”)  Searched via OVID on 16^th^ September 2020 and updated on 17^th^ November 2021. No date or language limits were imposed.  153 results |
| OpenGrey | (surgery OR "general surgery") AND (“cognitive stimulation” OR “cognitive rehabilitation” OR “cognitive training” OR “cognitive retraining” OR “cognitive re-training” OR “cognitive support” OR “memory function” OR “memory rehabilitation” OR “memory therapy” OR “memory aid*” OR “memory group*” OR “memory training” OR “memory retraining” OR “memory support” OR “memory stimulation” OR “memory strategy” OR “memory management”)  Searched on 16^th^ September 2020 and updated on 17^th^ November 2021. No date or language restrictions were imposed.  20 results |
| ProQuest | (surgery OR "general surgery") AND (“cognitive stimulation” OR “cognitive rehabilitation” OR “cognitive training” OR “cognitive retraining” OR “cognitive re-training” OR “cognitive support” OR “memory function” OR “memory rehabilitation” OR “memory therapy” OR “memory aid*” OR “memory group*” OR “memory training” OR “memory retraining” OR “memory support” OR “memory stimulation” OR “memory strategy” OR “memory management”)  Searched on 16^th^ September 2020 and updated on 17^th^ November 2021. No date or language restrictions were imposed.  15 results |
| Total | Database search identified 993 results |

**Table S2**

**Excluded studies and reasons for exclusion (n=5)**

| **Primary author / year** | **Full citation** | **Reason for exclusion** |
| --- | --- | --- |
| Abdullah (2017) | Abdullah HR, Lien VP, Ong HK, Er PL, Hao Y, Khan SA et al. Protocol for a single-centre, randomised controlled study of a preoperative rehabilitation bundle in the frail and elderly undergoing abdominal surgery. BMJ open 2017;**7**:e016815. | Protocol only. |
| Butz (2019) | Butz M, El Shazly J, Sammer G, Tschernatsch M, Kastaun S, Yenigün M *et al.* *Decreasing postoperative cognitive deficits after heart surgery: protocol for a randomized controlled trial on cognitive training*. Trials 2019;**20**:733-33. | Protocol only. |
| Humeidan (2015) | Humeidan ML, Otey A, Zuleta-Alarcon A, Mavarez-Martinez A, Stoicea N, Bergese S. *Perioperative Cognitive Protection-Cognitive Exercise and Cognitive Reserve (The Neurobics Trial): A Single-blind Randomized Trial*. Clinical Therapeutics 2015;**37**:2641-50. | Wrong outcome; cognitive function measured < 7 days. |
| King (2015) | King S, Green HJ. *Psychological Intervention for Improving Cognitive Function in Cancer Survivors: A Literature Review and Randomized Controlled Trial*. Frontiers in Oncology 2015;**5**. | Wrong population; not all patients in the intervention group had surgery / unable to separate data. |
| Vlisides (2019) | Vlisides PE, Das AR, Thompson AM, Kunkler B, Zierau M, Cantley MJ *et al.* *Home-based Cognitive Prehabilitation in Older Surgical Patients: A Feasibility Study*. Journal Of Neurosurgical Anesthesiology 2019;**31**:212-17. | Wrong outcome; cognitive function measured < 7 days. |

**Table S3**

Study characteristics of included studies

| **Study**  Primary author (year), country, design | **Design** (including single or multicentre) | **Study Population** | | | **Specific domains targeted**^a^ | **Timing of assessment(s)** | **Outcome of interest** | **Significant effect for intervention group**^b^ | **Effect size (**where reported) |
| --- | --- | --- | --- | --- | --- | --- | --- | --- | --- |
|  |  | **Type of surgery,**  **Number** (IG/CG) | **Mean age IG / CG**  years (SD) | **Gender**  IG / CG  (n, %) |  |  |  |  |  |
| Ajtahed (2019), Iran | Three arm quasi-experimental interventional trial, single centre | CABG,  25/22/25^c^ | 59.96 (5.20) /  57.95 (9.76) /  56.48 (12.73)^c^ | Male:  15 (60) / 14 (63.6) / 20 (80)  Female: 10 (40) / 8 (36.4)/ 5 (20)^c^ | Attention,  working memory,  inhibition. | Pre-intervention (after surgery), post intervention,  6-month follow up. | CPT | = | 0.05^f^ |
|  |  |  |  |  |  |  | Flanker test | = | 0.15 ^f^ |
|  |  |  |  |  |  |  | UFoVT | = | 0.12 ^f^ |
|  |  |  |  |  |  |  | Digit span (forward and backwards) | + | 0.69 ^f^ |
|  |  |  |  |  |  |  | QoL (SF-36) | + | 0.22 ^f^ |
| Carbone (2019), Italy | Pilot RCT (parallel), single centre | Partial or total arthroplasty of the knee,  18/16 | 69.50 (3.20) /  69.69 (4.01) | Male: 7 (38.9) / 9 (56.25)  Female: 11(61.1) / 7 (43.75) | Working memory. | Pre-intervention (before surgery),  Immediately post-intervention. | CWMST | + | 1.91 ^g^ |
|  |  |  |  |  |  |  | Digit span forward | + | 0.87 ^g^ |
|  |  |  |  |  |  |  | Digit span backwards | = | 0.53 ^g^ |
|  |  |  |  |  |  |  | RAVLT – immediate recall | = | 0.23 ^g^ |
|  |  |  |  |  |  |  | RAVLT – delayed recall | = | 0.11 ^g^ |
|  |  |  |  |  |  |  | CWMST – intrusion errors | + | 0.96 ^g^ |
|  |  |  |  |  |  |  | GDS | + | 0.43 ^g^ |
|  |  |  |  |  |  |  | HAD (anxiety subscale) | + | 0.54 ^g^ |
| Cheng (2012), Taiwan | Pilot RCT (parallel), single centre | TKR ± THR, 25/25 | 73.0 (6.3) /  72.6 (5.1) | Male: 4 (16) /  1 (4)  Female:  21(84) / 24 (96) | Global cognitive function. | Pre-intervention (before surgery),  Post-intervention (discharge),  One month follow-up. | MMSE | + | Not reported |
| de Tournay-Jetté (2012), Canada | Quasi-experimental^d^ | CABG,  13/13/18^e^ | 69.92 (3.93) /  70.85 (4.51) /  70.89 (4.44)^d^ | Male:  10 (76.9) / 8 (61.5) 17 (94.4)^d^  Female:  3 (32.1) / 5 (38.5) / 1 (5.6) ^d^ | Attention, memory | Pre-intervention (1 month after surgery),  2 months post operatively (between intervention sessions, experimental groups only),  Follow-ups: 3 and 6 months. | Logical memory subset of the Rivermead battery | = | Not reported |
|  |  |  |  |  |  |  | RAVLT | + |  |
|  |  |  |  |  |  |  | WAIS-R digit symbol | + |  |
|  |  |  |  |  |  |  | TMT-A | = |  |
|  |  |  |  |  |  |  | TMT-B | = |  |
|  |  |  |  |  |  |  | Stroop | + |  |
|  |  |  |  |  |  |  | Verbal fluency | = |  |
| Eryomina (2015), Russia | Two arm quasi-experimental interventional trial, single centre | CABG,  37/37 | 60.0 (6.42) / 60.5 (6.42) | Not stated. | Attention, visual memorization, countdown, visuospatial stimulation. | Pre-intervention (after surgery),  follow-up: 12 days. | MMSE | + | Not reported |
|  |  |  |  |  |  |  | FAB | = |  |
|  |  |  |  |  |  |  | Clock drawing test | = |  |
|  |  |  |  |  |  |  | Schulte’s tables | = |  |
|  |  |  |  |  |  |  | 10 word memory task | + |  |
|  |  |  |  |  |  |  | Spontaneous visual memorisation | + |  |
|  |  |  |  |  |  |  | Spontaneous visual memorisation (delayed recall) | + |  |
|  |  |  |  |  |  |  | Verbal fluency test | + |  |
|  |  |  |  |  |  |  | Mattis dementia rating scale | = |  |
|  |  |  |  |  |  |  | Counting down | = | Not reported |
| Kulason (2018),  Japan | Pilot RCT (parallel), single centre | Lung surgery, 6/4 | 69 (6.96) /  68.75 (4.27) | Male:  3 (50) /  1 (25)  Female:  3 (50) / 3 (75) | Global cognitive function, executive functions. | Pre-intervention ,  3-month follow-up. | MMSE-J | = |  |
|  |  |  |  |  |  |  | FAB | = |  |
|  |  |  |  |  |  |  | FAB motor-programming sub-score | + |  |
|  |  |  |  |  |  |  | CBB | = |  |
|  |  |  |  |  |  |  | GHQ-12 | = |  |
|  |  |  |  |  |  |  | GDS | + |  |
|  |  |  |  |  |  |  | QoL-5 | + |  |
| O’Gara (2020), USA | RCT (parallel) feasibility, single centre | Cardiac surgery (CABG ± valve), 20/20 | 70 (6) /  69 (7) | Male:  14 (70) /  15 (75)  Female:  6 (30) / 5 (25) | Memory, attention, problem solving, flexibility, processing speed. | Pre-intervention (enrolment),  preoperative (on the day of surgery),  day of discharge,  Follow-ups: 1, 3, 6 months postoperatively. | t-MoCA | = | Not reported |
| Saleh (2015), China | RCT (parallel), single centre | Gastro-intestinal surgery,  69/72 | 71 (6) /  70 (6) | Male:  36 (52.2) / 38 (52.8)  Female:  33 (47.8) / 34 (47.2) | Memory. | Pre-intervention (before surgery),  Follow-up: one week after surgery. | JLO | = | Not reported |
|  |  |  |  |  |  |  | Digit span (forwards and backwards) | = |  |
|  |  |  |  |  |  |  | BVMT-R | + |  |
|  |  |  |  |  |  |  | SDMT | + |  |
|  |  |  |  |  |  |  | BVMT-R delayed recall test | = |  |
|  |  |  |  |  |  |  | BVMT-R discrimination index | = |  |
|  |  |  |  |  |  |  | TMT-A | = |  |
|  |  |  |  |  |  |  | TMT-B | = |  |
|  |  |  |  |  |  |  | Verbal fluency test | = |  |
| Song (2019), China | RCT (parallel) feasibility, single centre | Lung transplant, 23/23 | 65.0 (6.2) /  66.8 (4.7) | Male:  15 (65.2) /  17 (73.9)  Female:  8 (34.8) / 6 (26.1) | Attention, information processing speed, working memory. | Pre-intervention (4 weeks after surgery),  immediately after intervention,  12-week follow-up. | Digit span forward | + | Not reported |
|  |  |  |  |  |  |  | Digit span backwards | = |  |
|  |  |  |  |  |  |  | Digit symbol | = |  |
|  |  |  |  |  |  |  | TMT-A | = |  |
|  |  |  |  |  |  |  | TMT-B | = |  |
|  |  |  |  |  |  |  | Verbal fluency test | + |  |
|  |  |  |  |  |  |  | Word recognition test | = |  |

^a^ as reported by the authors of the studies; ^b^ at final follow-up unless indicated otherwise; ^c^ IG / AC / CG, ^d^ Participants were assigned to one of three cohorts, then two patients from each cohort were randomly assigned to one of the three experimental conditions (control group, attention training followed by memory training (A-M), memory training followed by attention training (M-A)]; ^e^ attention-memory (A-M) group / Memory-attention (M-A) group / control; ^f^ partial eta squared (η^2^); ^g^ short term net effect sizes, determined using the formula: [post-test for trained group – pretest for trained group) – (post-test for the control group – pretest for the control group)] / (pooled standard deviation of the adjusted difference). All net effects sizes were coded so that positive values denote a better performance.

**Effect of intervention**: + positive effect for intervention group, = no difference between groups, - negative effect for intervention group

**Abbreviations:** AC – active control; BVMT-R - Brief Visuospatial Memory Test – Revised; CBB - Cogstate Brief Battery; CG – control group; CPT – Continuous performance test; CWMST - Categorization Working Memory Span Task; FAB – frontal assessment battery; GDS – geriatric depression scale; GHQ-12 – general health questionnaire-12; HADS – hospital anxiety and depression scale; IG – intervention group; JLO - Categorization Working Memory Span Task; MMSE - Mini Mental State Exam; MMSE-J - Mini Mental State Exam – Japanese; QoL – quality of life; QoL-5 – quality of life scale-5; RAVLT - Rey Auditory and Verbal Learning Test; SDMT - Symbol Digit Modalities Test; THR – total hip replacement; TKR – total knee replacement; t-MoCA – Telephonic Montreal Cognitive Assessment; TMT-A – trail making test part A; TMT-B – trail making test part B; UFoVT – useful field of view test; WAIS-R - Wechsler Adult intelligence Scale – Revised.

**Table S4**

Yeas of education (intervention groups)

| **Study**  *Primary author* *(year)* | **Years of education (intervention group)**  n (%) or years (SD) |
| --- | --- |
| Ajtahed (2019) | Primary school 5 (20%)  High school 14 (56%)  University 6 (24%) |
| Carbone (2019) | Education 7.17 (1.38) |
| Cheng (2012) | Education 5.3 (4.1) |
| de Tournay-Jetté (2012) | Education 10.78 (5.23) |
| Eryomina (2015) | Secondary 10 (27.1%)  Vocational secondary 16 (43.2%)  Higher 11 (29.7%) |
| Kulason (2018) | Not stated |
| O’Gara (2020) | High school graduate or equivalent 1 (5%)  Some college, associate’s degree 4 (20%)  Bachelor’s degree 8 (40%)  Masters’s degree 5 (25%)  Doctoral degree 2 (10%) |
| Saleh (2015) | Education 5.7 (2.0) |
| Song (2019) | Education < 6 years 11 (23.9%)  Education 6-12 years 27 (58.7%)  Education > 12 years 8 (17.4%) |

**Table S5**

Baseline Cognition (inclusion / exclusion criteria)

| **Study**  *Primary author* *(year)* | **Baseline cognition** | |
| --- | --- | --- |
|  | **Inclusion** | **Exclusion** |
| Ajtahed (2019) | Not stated |  |
| Carbone (2019) | MMSE ≥26 |  |
| Cheng (2012) | Not stated |  |
| de Tournay-Jetté (2012) |  | MMSE <24 |
| Eryomina (2015) | Not stated |  |
| Kulason (2018) | Not stated |  |
| O’Gara (2020) |  | MOCA < 10 |
| Saleh (2015) |  | MMSE ≤ 23 before surgery |
| Song (2019) |  | MOCA ≤26  MMSE <24/30 |
